# Severe periodontitis and congenital cytomegalovirus: Cleft lip and cleft palate

**DOI:** 10.1111/prd.12606

**Published:** 2024-09-11

**Authors:** Jørgen Slots, Vanessa Slots

**Affiliations:** ^1^ Division of Periodontology and Diagnostic Sciences University of Southern California School of Dentistry Los Angeles California USA; ^2^ M Health Fairview Woodbury Minnesota USA

**Keywords:** cleft lip and cleft palate, congenital diseases, cytomegalovirus, periodontal treatment, periodontitis

## Abstract

Severe periodontitis lesions can harbor several hundred‐thousand copies of active cytomegalovirus, and this paper proposes that cytomegalovirus in maternal periodontitis can infect the fetus. Cleft lips and palates may be oral examples of congenital cytomegalovirus infection. Anti‐cytomegalovirus periodontal treatment is indicated for high‐risk women who exhibit severe periodontitis and weakened immune system and are contemplating pregnancy or are in the first trimester of pregnancy.

## 1 INTRODUCTION

Cytomegalovirus is a herpesvirus that infects people of all ages.^1^ In the United States, cytomegalovirus has infected nearly 1 in 3 children by age 5, and over half of adults by age 40. 1 in 100 women contract a cytomegalovirus primary/active infection during pregnancy. A pregnant woman can potentially transmit cytomegalovirus virions across the placenta and infect the fetus. About 1 in 200 of newborn babies show congenital cytomegalovirus infection (cytomegalovirus DNA in the newborn's urine or saliva within 2–3 weeks of life), and 1 in 5 infants born with congenital cytomegalovirus infection experience health problems at birth or later in life.

Primary cytomegalovirus infection of the mother during the periconceptional period or the first trimester of pregnancy may result in physical and neurologic sequelae in 20%–30% of infants versus in 1% if acquired later in pregnancy.^2^, ^3^ Congenital cytomegalovirus may cause intrauterine growth restriction, prematurity, microcephaly, jaundice, petechiae, hepatosplenomegaly, periventricular calcifications, chorioretinitis, hepatitis, and pneumonitis. Symptomatic neonates are at risk of developing neurologic impairment, including sensorineural hearing loss (most common), visual disturbances, and intellectual disability. Hearing loss may be detected soon after birth or develop later in childhood. Particularly severe congenital cytomegalovirus infection may result in abortion, stillbirth, or postnatal death (30%).

Severe/progressive periodontitis lesions^4^ and acute endodontic abscesses^5^, ^6^ can harbor hundred‐thousands of active (lytic) cytomegaloviruses and constitute a major reservoir of cytomegalovirus outside the hematopoietic compartment.^7^ Herpesviruses enter periodontal lesions as latent DNA embedded in inflammatory cells, but metabolic products of coresident periodontopathic bacteria^8^ or hormonal changes during pregnancy may activate periodontal herpesviruses. Periodontal cytomegalovirus virions have ready access to the systemic circulation and via transplacental transmission may infect the fetus and cause clinical disease, especially in immunodeficient mothers. Active cytomegalovirus in severe periodontitis may be an important nidus for congenital disorders. Oncogenic viruses and bacteria can also inhabit periodontitis lesions,^9^, ^10^ underscoring the seriousness of life‐threatening diseases potentially originating from the mouth.

The wide‐ranging systemic morbidity of congenital cytomegalovirus makes it reasonable to assume that pathogenic disorders also exist in the oral cavity. This paper considers oral diseases that may have a congenital cytomegalovirus etiology and, to help prevent congenital cytomegalovirus infection, proposes an anti‐cytomegalovirus, noninvasive, and inexpensive periodontal treatment for women who exhibit severe periodontitis and weakened immune systems and are contemplating pregnancy or are already pregnant.

## 2 OROFACIAL BIRTH DEFECTS

Orofacial birth defects may be among the most common physical disorders in the United States, with 1 in 700 of newborns suffering from cleft lip and/or cleft palate.^11^ Cleft lip is most common in individuals with Native American and Asian ancestry and least common in descents from Africa. Cleft palate occurs equally in all ethnic groups. Orofacial clefts may be due to infectious, genetic and environmental determinants, but the etiopathogenesis is inconclusive in many cases.

Severe birth malformations of the face and mouth result from disturbances in the embryonic development. The face begins to form by week 5 and the lip is completed at week 5 post‐conception. The palate develops between weeks 7 and 11 dependent on the growth rate of the head. Lips can show unilateral and bilateral clefts, and lip and palate clefts can both appear as partially or fully formed defects. Orofacial clefts, especially cleft lip, can be diagnosed by routine ultrasound during pregnancy.

Orofacial birth defects may hamper breathing, hearing, and speech and language development. Surgically repair of cleft lip usually takes place within the first 12 months of life and cleft palate within the first 18 months of life. Some children may need therapy into adulthood. Submucosal cleft palate and bifid uvula might not be diagnosed at birth, and several individuals born with orofacial clefts may eventually need speech therapy and prosthodontic or orthodontic treatment.

## 3 CYTOMEGALOVIRUS CAUSING OROFACIAL BIRTH DEFECTS

Research on the specific pathogenic mechanisms of orofacial birth defects is slowly making progress.^12^, ^13^ Cytomegalovirus active infection has been related to orofacial clefts^14^, ^15^ and maternal periodontitis, which is likely to contain cytomegalovirus, has been linked to preeclampsia,^16^ premature birth (<37 weeks of pregnancy),^17^ and low birthweight infants (<2500 g).^17^ Cytomegalovirus that infects odontogenic cells may interfere with normal tooth formation,^18^, ^19^ and congenital/perinatal cytomegalovirus infection can alter the morphology of developing teeth^20^ and dental root surfaces,^4^ predisposing children and adolescents to rapidly advancing periodontitis.^4^ Congenital/perinatal cytomegalovirus infection may also cause hypodontia (congenitally missing teeth), although scientific confirmation is lacking.

## 4 PREVENTIVE PERIODONTAL THERAPY FOR CONGENITAL CYTOMEGALOVIRUS

An evidence‐based therapy to prevent mother‐to‐fetus transmission of cytomegalovirus was unavailable until recently. In 2020, Shaha‐Nissan et al.^21^ showed that high dosage of valacyclovir (8 g daily from enrolment until amniocentesis at 21/22 gestational weeks) reduced the vertical cytomegalovirus transmission rate by 70% in women who had acquired a primary cytomegalovirus infection during the periconceptional period or in the first trimester of pregnancy. Valacyclovir inhibits herpesvirus DNA polymerase and is effective only against active herpesviruses. Recent systematic review,^22^ narrative review,^23^ and large multicenter study^24^ have reaffirmed the value of valacyclovir for secondary prevention of congenital cytomegalovirus. Only 24 of 205 women experienced mild to moderate reversible symptoms from the valacyclovir therapy.^24^ Valacyclovir is currently the only medication proven scientifically to decrease cytomegalovirus transmission from mother to child.

Periodontal therapy may help reduce congenital cytomegalovirus infections. Kumar et al.^25^ treated aggressive periodontitis and found after 3 months a 37% reduction in cytomegalovirus serum IgG antibodies, indicating severely diseased periodontium carries a significant segment of cytomegalovirus total body load. To minimize adverse effects on the mother and fetus, the treatment suggested here focuses on neutralizing active cytomegalovirus in the periodontium during the high‐risk 2–3‐month period before conception and the first trimester of pregnancy. The truncated treatment includes systemic valacyclovir (1 g BID on day 1, and 500 mg BID on days 2–7) and ultrasonic periodontal scaling with 0.1% sodium hypochlorite cooling fluid but omits periodontal surgery and anaerobic antibiotics. Valacyclovir is already used in the treatment of severe periodontitis.^26^ Patients are requested to perform traditional oral hygiene procedures, daily subgingival irrigation with freshly prepared 0.1% sodium hypochlorite using Waterpik Pik Pocket™ (Fort Collins, CO, USA) or similar delivery device, and twice weekly oral rinsing for 30 s with 0.1% sodium hypochlorite. Saliva‐based cytomegalovirus quantitative PCR assays may assess therapeutic progress.^27^

The American Dental Association Council on Dental Therapeutics approved sodium hypochlorite solutions up to 0.25% as a “mildly antiseptic mouthwash” for direct application onto human mucous membranes.^28^ Sodium hypochlorite occurs naturally in neutrophils and macrophages and is safe^29^ and available as medical grade or regular household bleach. A 0.1%–0.2% sodium hypochlorite solution can be obtained by adding one teaspoon (5 mL) of 6%–9% bleach to a large glass (250 mL/8.5 oz) of water.

## 5 SUMMARY

Contemporary periodontology emphasizes both prevention of tooth loss and reduction of systemic disease risks.^30^, ^31^ This paper suggests adding congenital cytomegalovirus to the long list of serious diseases that are linked to severe periodontitis. Anti‐cytomegalovirus periodontal treatment is also recommended to potentially reduce the incidence of congenital diseases. Research is obviously needed to delineate the role of severe periodontitis in congenital cytomegalovirus, but it seems unduly restrictive to await confirmative scientific evidence before undertaking periodontal treatment of current pregnant women who are at high risk for congenital cytomegalovirus infection and complications. An additional consideration, aside from potentially preventing congenital cytomegalovirus, the present low‐cost therapy would improve the periodontal health status and decrease the risk for tooth loss during the pregnancy period.

## CONFLICT OF INTEREST STATEMENT

The authors declare no conflict of interest.

## Data Availability

Data sharing not applicable to this article as no datasets were generated or analysed during the current study.
